# Using Machine Learning to Derive Just-In-Time and Personalized Predictors of Stress: Observational Study Bridging the Gap Between Nomothetic and Ideographic Approaches

**DOI:** 10.2196/12910

**Published:** 2019-04-26

**Authors:** Alan Rozet, Ian M Kronish, Joseph E Schwartz, Karina W Davidson

**Affiliations:** 1 Center for Behavioral Cardiovascular Health Columbia University Irving Medical Center New York, NY United States; 2 Feinstein Institute for Medical Research Northwell Health New York, NY United States

**Keywords:** ecological momentary assessment, machine learning, stress-behavior pathway, personal informatics, self-quantification, exercise, weather, just-in-time interventions

## Abstract

**Background:**

Investigations into person-specific predictors of stress have typically taken either a population-level nomothetic approach or an individualized ideographic approach. Nomothetic approaches can quickly identify predictors but can be hindered by the heterogeneity of these predictors across individuals and time. Ideographic approaches may result in more predictive models at the individual level but require a longer period of data collection to identify robust predictors.

**Objective:**

Our objectives were to compare predictors of stress identified through nomothetic and ideographic models and to assess whether sequentially combining nomothetic and ideographic models could yield more accurate and actionable predictions of stress than relying on either model. At the same time, we sought to maintain the interpretability necessary to retrieve individual predictors of stress despite using nomothetic models.

**Methods:**

Data collected in a 1-year observational study of 79 participants performing low levels of exercise were used. Physical activity was continuously and objectively monitored by actigraphy. Perceived stress was recorded by participants via daily ecological momentary assessments on a mobile app. Environmental variables including daylight time, temperature, and precipitation were retrieved from the public archives. Using these environmental, actigraphy, and mobile assessment data, we built machine learning models to predict individual stress ratings using linear, decision tree, and neural network techniques employing nomothetic and ideographic approaches. The accuracy of the approaches for predicting individual stress ratings was compared based on classification errors.

**Results:**

Across the group of patients, an individual’s recent history of stress ratings was most heavily weighted in predicting a future stress rating in the nomothetic recurrent neural network model, whereas environmental factors such as temperature and daylight, as well as duration and frequency of bouts of exercise, were more heavily weighted in the ideographic models. The nomothetic recurrent neural network model was the highest performing nomothetic model and yielded 72% accuracy for an 80%/20% train/test split. Using the same 80/20 split, the ideographic models yielded 75% accuracy. However, restricting ideographic models to participants with more than 50 valid days in the training set, with the same 80/20 split, yielded 85% accuracy.

**Conclusions:**

We conclude that for some applications, nomothetic models may be useful for yielding higher initial performance while still surfacing personalized predictors of stress, before switching to ideographic models upon sufficient data collection.

## Introduction

Deeper knowledge of the day-to-day effects of both weather and physical activity on stress can be valuable for creating personalized stress-reduction interventions on a just-in-time basis. Previous investigations have often focused on a nomothetic approach, pooling data to identify influential features across individuals [1,2]. However, this approach typically has a drawback: Insight into any particular individual is limited due to heterogeneous effects of factors on individual-level stress and may not be generalized due to biological variability or overfitting [3]. For example, hot weather may reduce stress for some participants, but increase stress for others. To remedy this, some have taken an ideographic approach, developing personalized stress-prediction models for each person [4-6].

Although this ideographic strategy resolves the issue of achieving per-individual insights, it does so by discarding potentially useful data from other individuals. The ideographic approach also requires the acquisition of at least some data about a given individual before making predictions for that individual. Identifying predictors of potentially low-frequency events such as occurrences of high stress may require substantial data collection before the ideographic model becomes sufficiently robust to confidently identify predictors. Thus, prior to obtaining reliable predictors of stress, a given individual may have to complete a long period of self-tracking, which may not be acceptable to some individuals.

Here, we first compared individual-level predictors of stress identified through nomothetic models to those identified in ideographic models. We next explored the accuracy of a model-switching paradigm that begins with a nomothetic model and progressively changes to an ideographic model for data for the individual accumulate. We hypothesized that beginning with a nomothetic model would maximize accuracy during the early phase of data collection (referred to as a “warm start”) and subsequently, switching to ideographic modeling for higher personalization and performance would be the most effective approach to maximizing accuracy throughout data collection.

## Methods

### Overview

This paper used the data collected in an observational study of 79 participants who were followed for up to 1 year, starting between January and July 2014; the study continuously and objectively monitored the physical activity of the participants by actigraphy and the perceived stress rating through ecological momentary assessment (EMA) reports on a mobile app [6]. Participants were healthy individuals, aged 18 years or older, who responded to fliers posted throughout the buildings of Columbia University Medical Center (New York City, NY) and who, on phone screening, reported only intermittent engagement in exercise and having access to a personal computer and iOS or Android smartphone. Individuals with significant medical comorbidities, occupational work demands requiring rigorous physical activity, or inability to read and speak English were excluded. During a baseline interview, demographic characteristics including age, sex, race, ethnicity, education, partner status, and living situation were collected.

### Measures

Stress was measured using an end-of-day text message survey on the participant’s own iPhone or Android phone, with the question “Overall, how stressful was your day?” Each evening, the participant was also asked, “Overall, how stressful do you think tomorrow will be?” Each morning, the participant responded to the questions “How stressful do you expect today to be?” and “How likely are you to exercise today?” All responses were rated on a scale from 0 (not at all) to 10 (extremely). All surveys were administered using Qualtrics software (Qualtrics, Seattle, WA). Two participants’ data were excluded for almost no variance in the self-reported stress ratings, leading to a total of 77 participants for the analysis.

Physical activity was measured using a wrist-worn Fitbit (Fitbit, Inc, San Francisco, CA) to track daily physical activity, including the steps taken, calories burned, and intensity of physical activity for each minute of the day. Participants were instructed to sync and charge the device every 5 to 7 days. In this analysis, a bout of “exercise” was defined as any consecutive 30-minute period within which 24 or more minutes of moderate- or vigorous-intensity activity was performed. We followed the recommendations of Ward et al [7] regarding best practices for the use of accelerometer data in research on physical activity. Specifically, physical activity guidelines recommend exercising for at least 30 minutes a day while accommodating interruptions. Further, when analyzing accelerometer data, the conventional approach is to quantify exercise in bouts of 10 minutes with allowances for 2 minutes of interruption (ie, total exercise for 8 of 10 min). Extrapolating the definition based on 10 to 30 minutes of activity, this yielded 24 of 30 minutes of activity. Software was written to determine, for each day, whether there was any 30-minute period within which at least 24 minutes of moderate or vigorous activity was performed; this was our objectively assessed measure of a 30-minute period of exercise. Days in which the Fitbit device was worn for fewer than 10 hours were excluded from all analyses.

External and environmental variables, including temperature (high, low, average, and range), hours of daylight, precipitation, and day of the week, were retrieved from the meteorological station in Central Park (New York City, NY); these data are made publicly available by the National Oceanic and Atmospheric Administration’s National Center for Environmental Information.

### Statistical Analysis

We developed models for stress rating using neural network, decision tree, and linear approaches across all participants, as well as participant-specific decision trees. Each model predicted a person’s self-reported stress rating (range, 0-10), using the previous 3 days of weather, self-reported stress, and actigraphy data. Because the previous 3 days were used as inputs, periods missing a stress rating were excluded.

The continuous prediction for stress was then converted into a binary classification as either above or below a participant’s median self-reported stress value in the training set. We chose this approach, because if the stress rating was left as a continuous value, it was not clear at what rating an intervention should be initiated. Further, using the participant’s median as a reference slightly adjusts for participants who did not utilize the full range of the 11-point scale. In this way, the high-stress rating was individualized for each participant. The Keras Python library [8] was used to train neural network models, and the scikit-learn Python library [9] was used to train linear and decision tree-type models. Dropout, a regularization method for neural networks, was also used in the neural network models during training to decrease overfitting.

Model performance was compared based on regression (mean absolute error) and classification (area under the curve [AUC], F_1_ score, accuracy) error in predicting stress self-report. Feature importances were also compared between models. The AUC of a classifier is equivalent to the probability that the classifier will rank a randomly chosen positive instance

higher than a randomly chosen negative instance [10]. A model with an AUC closer to 1 is generally better. An F_1_ score is the harmonic mean of a model’s precision and recall, with values between 0 and 1, in which values closer to 1 are better. Model selection was performed through exhaustive grid search of the corresponding hyperparameters for each model using 3-fold cross-validation and optimizing on the mean absolute error.

For the ideographic decision trees, the Gini importance, as implemented in scikit-learn, was used to derive the significance of each input variable for each participant. Layer-wise relevance propagation (LRP) [11] was used to interpret our neural network models. LRP propagates the relevance of each input variable back through the network from a specific prediction. In other words, for this dataset, a participant with 30 valid days in the dataset would yield 30 sets of LRP relevance scores, each set having one score for each input variable. These scores can be positive or negative in a similar fashion as linear coefficients, and the greater the magnitude of the score, the more that particular variable increased or decreased that particular prediction for stress rating. LRP was used in favor of other methods such as Deep Learning Important Features (DeepLIFT) and Integrated Gradients, because it does not require a baseline or reference value. However, LRP can be viewed as an approximation of DeepLIFT when bias terms are 0 and the reference values are set to 0 [12]. This, in turn, approximates Shapley values [13], which is another method of interpreting neural network output. As such, to leverage the visualizations for Shapley values built in the Shapley Additive Explanations Python library and to add an additional method of visual interpretation, bias terms for the neural net were locked to 0; this did not impact model performance.

The following variables were used for the ideographic models: the number of hours with ≥10 activities, total exercise duration in minutes during bouts of exercise with ≥24 minutes of activity out of 30 minutes, the number of exercise bouts with ≥24 minutes of activity out of 30 minutes, the binary presence of any exercise bout of ≥24 minutes of activity out of 30 minutes, the total number of exercise bouts, the binary presence of any exercise bout, total exercise duration, stress rating, minimum outdoor temperature, maximum outdoor temperature, average outdoor temperature, total daylight minutes, and total precipitation. Several person-level variables were included in the nomothetic models that were not included in the per-participant models, as they would have been static for a given participant and would not have contributed to performance. These included height, weight, age, and sex. The inclusion of these variables in the nomothetic models slightly improved their performance.

Additionally, all models were trained with varying training set sizes to test the hypothesis that ideographic models may be more reliable at large-enough training set sizes for each participant. Training set sizes ranged from 30% to 80% of valid days for each participant. For example, the first 30% of days in which a participant documented his or her stress was used to train a model predicting the subsequent 70% of days. The results for an 80% train/20% test split are highlighted here, with a total of 4050 training set samples and 678 testing set samples available to all nomothetic models.

All models were compared to a baseline model that simply predicted the median of the stress self-report values from a participant’s training set, for all test samples.

## Results

### Overview

The average age of the participants was 32 years (range, 20-58 years), with a height of 169 cm and weight of 75 kg. The study sample included 34 men and 43 women in the dataset. Table 1 depicts the basic descriptive statistics on the age, height, and weight of our set of participants.

**Figure 1:**
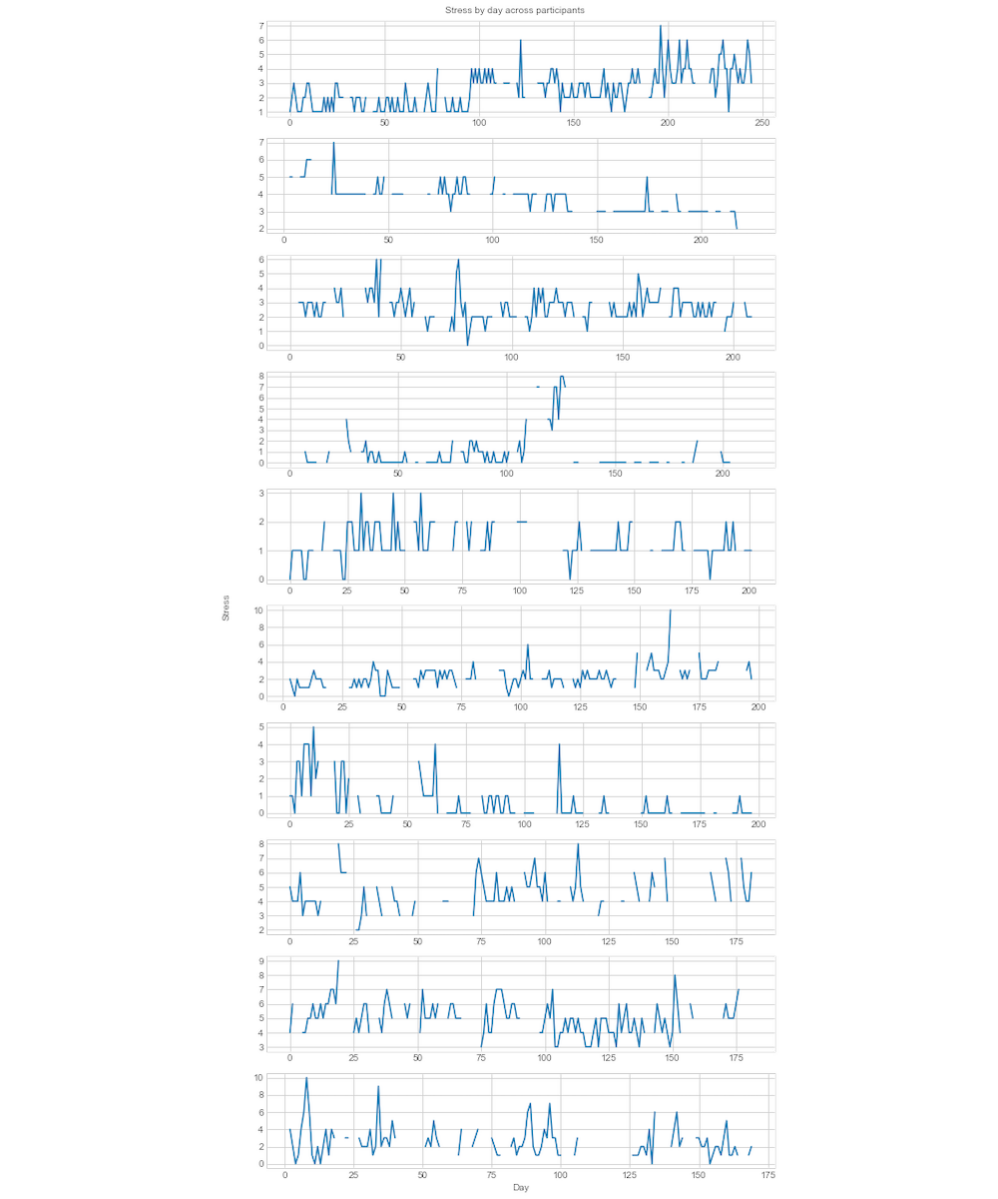
Plots of stress ratings for the participants with the greatest amount of responses.

The pattern of stress ratings differed significantly among participants. Figure 1 shows plots of stress by day for the 10 participants with the greatest number of valid responses in the dataset. Days without responses are shown without points plotted.

**Table 1:** Basic summary statistics for the participants (N=77).

Statistic	Mean (SD)	Min	25%^a^	50%^a^	75%^a^	Max
Age (years)	31.62 (9.42)	20	24	27	38	58
Height (cm)	168.71 (8.49)	149.4	162	168	176	187
Weight (kg)	75.68 (17.46)	43.3	62.5	74.7	85.5	138.6

^a^Quartile ranges.

**Table 2:** Summary statistics and count of stress ratings, grouped by occurrence of missing stress ratings for the 3 days before the date of the predicted stress rating.

Response	Count	Mean stress (SD)	Min	25%^a^	50%^a^	75%^a^	Max
000	1728	3.60 (2.58)	0	2	3	6	10
001	1077	3.40 (2.57)	0	1	3	5	10
010	292	3.36 (2.63)	0	1	3	5	10
011	973	3.31 (2.51)	0	1	3	5	10
100	973	3.16 (2.54)	0	1	3	4	10
101	239	3.35 (2.63)	0	1	3	5	10
110	889	3.17 (2.48)	0	1	3	4	10
111	4060	2.93 (2.35)	0	1	3	4	10

^a^Quartile ranges.

**Table 3:** Percentage of stress ratings grouped by the occurrence of missing stress ratings for the 3 days before the date of the predicted stress rating. Each column corresponds to the value of the stress rating, and each cell represents what percentage of stress ratings had that value and response pattern.

Response	0	1	2	3	4	5	6	7	8	9	10
000	11.92	12.21	14.47	14.47	15.74	5.73	8.91	8.39	4.40	1.50	2.26
001	13.65	12.26	16.06	16.34	12.91	6.41	8.73	6.41	2.79	1.58	2.88
010	17.47	9.93	13.36	17.12	13.70	6.16	7.88	7.19	3.42	0.00	3.77
011	14.59	11.51	14.59	20.45	11.51	7.09	7.71	5.96	3.19	0.41	2.98
100	17.57	13.05	13.77	14.80	16.14	6.06	6.37	6.37	2.26	0.72	2.88
101	17.57	10.88	13.39	15.90	13.81	5.02	6.69	9.21	5.02	0.42	2.09
110	16.20	11.25	15.75	18.67	14.40	5.29	7.31	5.29	2.25	0.79	2.81
111	15.62	15.12	17.02	18.92	13.42	5.91	4.98	3.89	2.09	0.64	2.39

Table 2 shows the mean stress rating grouped by the occurrence of missing stress ratings for the 3 days before the date of the predicted stress rating. The response column describes the occurrence of stress ratings: 000 represents no stress ratings in any of the 3 days, 001 represents presence of only one stress rating recorded just before the predicted day, and 111 represents presence of all three stress ratings. Periods missing a stress rating were more often followed by a higher stress rating than periods that were not missing any stress ratings.

Table 3 shows the percentage of stress ratings grouped by the occurrence of missing stress ratings for the 3 days before the date of the predicted stress rating. The frequency of high-stress ratings is slightly higher for response patterns with missing stress ratings.

### Accuracy of Nomothetic Versus Ideographic Models

With an 80% training set/20% testing set split, the nomothetic recurrent neural network model AUC was 74.20% and the F_1_ score was 79.21%. In addition, the per-participant decision tree AUC was 0.67 and the F_1_ score was 0.83. The full results can be found in Multimedia Appendix 1. Nomothetic models, on an average, outperformed ideographic models for shorter training set sizes. As the training set size increased (ie, allowing for longer duration of assessments), group-level model performance generally increased across all models, and the nomothetic and ideographic model performances converged. However, when separately applying models to individual participants, ideographic models generally outperformed the nomothetic ones once the days with valid data exceeded 50 days.

Table 4 lists performance metrics for each model, grouped by training set proportion and model used. Precision, recall, F_1_ score, AUC, and accuracy are performance metrics. Training set size ranges from 30% to 80%. “Ideographic decision tree over 100 days” represents the performance metrics, only for participants who had at least 100 valid days, which is the same for the 50-day model.

**Table 4:** Performance metrics across all models, grouped by training set size.

Average number of days for training set size	Precision (%)	F_1_ score (%)	Area under the curve	Accuracy (%)	Training set size (%)	Model
16.7215	74.22	71.95	53.39	60.69	30.00	Ideographic Decision Tree
16.7215	75.76	72.63	54.40	61.53	30.00	Ideographic Decision Tree Over 50 Days
16.7215	76.61	71.86	52.73	60.21	30.00	Ideographic Decision Tree Over 100 Days
16.7215	77.80	78.77	62.88	69.75	30.00	Random Forest
16.7215	78.83	78.72	64.25	70.10	30.00	Gradient Boosted Decision Tree
16.7215	79.90	77.93	65.32	69.69	30.00	Recurrent Neural Network
16.7215	81.09	72.69	64.71	65.18	30.00	Baseline
16.7215	81.20	81.03	68.21	73.36	30.00	Elastic Net
16.7215	82.41	77.39	68.00	70.01	30.00	Neural Network
21.7595	75.35	72.40	54.44	61.37	40.00	Ideographic Decision Tree Over 100 Days
21.7595	76.48	73.06	57.12	62.79	40.00	Ideographic Decision Tree
21.7595	76.96	72.96	56.32	62.37	40.00	Ideographic Decision Tree Over 50 Days
21.7595	77.87	77.09	62.08	67.96	40.00	Gradient Boosted Decision Tree
21.7595	78.15	79.23	63.16	70.25	40.00	Random Forest
21.7595	80.93	71.11	63.74	63.61	40.00	Baseline
21.7595	81.47	76.25	66.23	68.47	40.00	Recurrent Neural Network
21.7595	81.70	80.15	68.15	72.48	40.00	Elastic Net
21.7595	82.33	79.40	68.55	71.90	40.00	Neural Network
27.3291	75.76	75.36	52.41	63.47	50.00	Ideographic Decision Tree
27.3291	77.09	75.95	52.52	64.06	50.00	Ideographic Decision Tree Over 50 Days
27.3291	77.27	75.56	50.10	63.07	50.00	Ideographic Decision Tree Over 100 Days
27.3291	82.41	82.65	67.54	74.60	50.00	Random Forest
27.3291	82.45	81.94	67.29	73.80	50.00	Gradient Boosted Decision Tree
27.3291	84.02	82.71	69.79	75.15	50.00	Recurrent Neural Network
27.3291	84.81	76.31	67.89	68.57	50.00	Baseline
27.3291	85.84	83.89	72.73	77.00	50.00	Elastic Net
27.3291	88.76	83.09	75.69	76.79	50.00	Neural Network
32.7215	78.54	76.99	54.63	65.59	60.00	Ideographic Decision Tree
32.7215	78.85	77.45	53.40	65.77	60.00	Ideographic Decision Tree Over 100 Days
32.7215	79.41	77.69	54.87	66.34	60.00	Ideographic Decision Tree Over 50 Days
32.7215	85.90	83.73	69.13	75.75	60.00	Random Forest
32.7215	86.53	76.05	66.83	67.36	60.00	Baseline
32.7215	88.76	83.49	73.24	76.19	60.00	Gradient Boosted Decision Tree
32.7215	89.80	85.19	75.62	78.47	60.00	Elastic Net
32.7215	90.78	82.20	75.20	75.15	60.00	Recurrent Neural Network
32.7215	91.35	80.73	75.08	73.62	60.00	Neural Network
38.1646	80.69	80.90	62.87	71.65	70.00	Baseline
38.1646	84.52	82.50	67.17	74.09	70.00	Ideographic Decision Tree
38.1646	84.64	83.78	69.06	75.96	70.00	Ideographic Decision Tree Over 50 Days
38.1646	87.35	85.71	74.69	79.23	70.00	Random Forest
38.1646	87.83	83.81	74.23	77.08	70.00	Gradient Boosted Decision Tree
38.1646	88.57	84.21	75.36	77.72	70.00	Elastic Net
38.1646	88.60	88.99	76.76	83.25	70.00	Ideographic Decision Tree Over 100 Days
38.1646	89.16	80.69	74.07	73.88	70.00	Recurrent Neural Network
38.1646	89.57	82.76	75.69	76.28	70.00	Neural Network
43.2025	81.44	81.79	63.94	72.81	80.00	Baseline
43.2025	84.10	83.00	67.72	74.82	80.00	Ideographic Decision Tree
43.2025	88.31	90.07	76.80	84.62	80.00	Ideographic Decision Tree Over 50 Days
43.2025	89.00	84.20	75.63	77.71	80.00	Elastic Net
43.2025	89.00	83.43	75.18	76.80	80.00	Gradient Boosted Decision Tree
43.2025	89.25	84.70	76.22	78.35	80.00	Random Forest
43.2025	90.38	78.91	74.20	72.16	80.00	Recurrent Neural Network
43.2025	90.57	90.78	79.87	85.82	80.00	Ideographic Decision Tree Over 100 Days
43.2025	90.89	73.87	72.06	67.27	80.00	Neural Network

### Predictors of Stress

**Figure 2:**
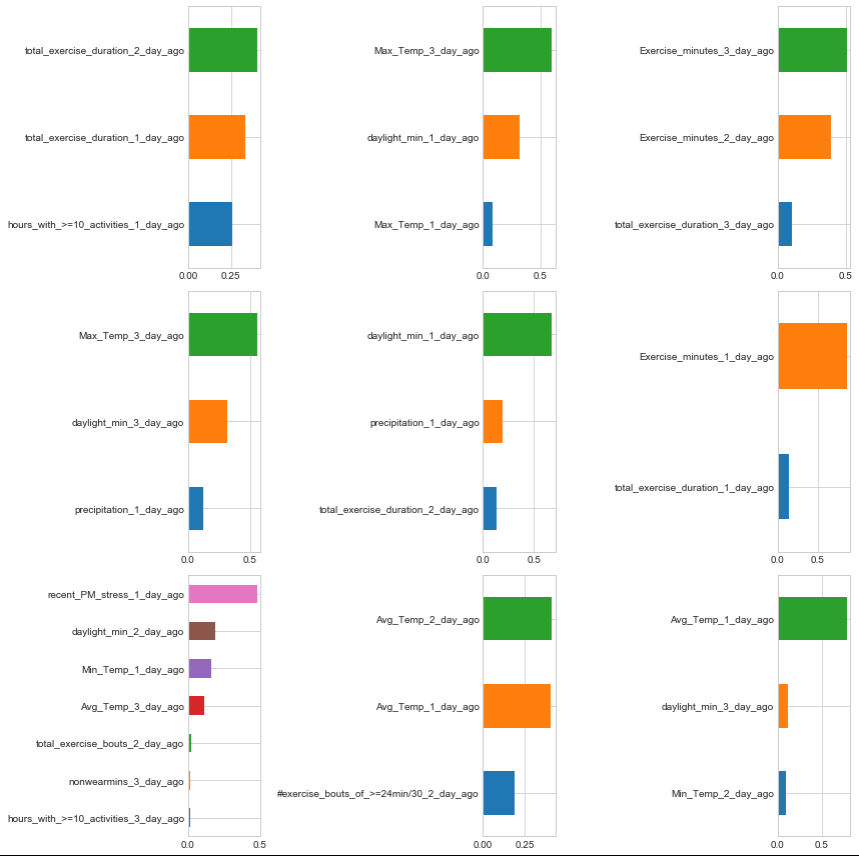
Most important predictors for the ideographic decision tree models for the 9 individuals with at least 100 valid days of data.

In our ideographic models, there was significant heterogeneity in the effect of features of weather and exercise. Figure 2 shows a series of horizontal bar plots, visualizing the Gini importance values, or the factors that significantly predicted day-to-day variability in stress rating for the 9 individuals with at least 100 valid days of data.

The most frequent important predictors for each participant included daylight minutes, temperature, and exercise behavior for the current or preceding 3 days. Often, variables that were assessed closer (temporally) to the stress rating being predicted exhibited greater importance.

**Figure 3:**
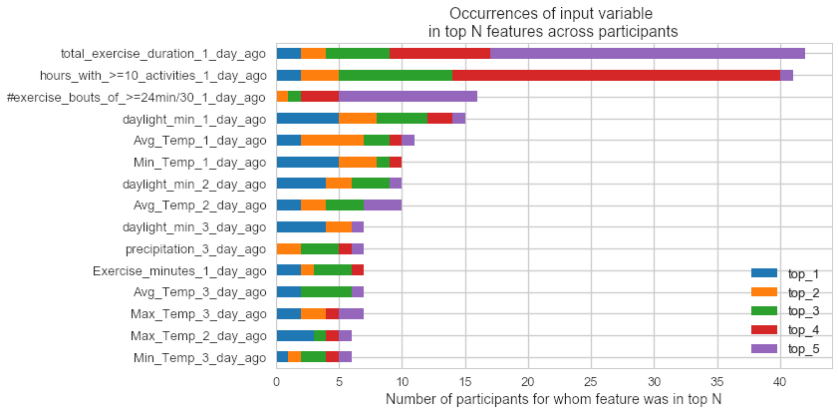
A horizontally stacked bar chart of occurrences of the most frequently appearing predictor variables, and how often they ranked in the top 5 spots across participants’ predictor variable importance rankings from the ideographic models.

Viewing feature importance in aggregate for all the participants in the dataset, the Gini importance values from their corresponding decision tree model were sorted and then ranked. Thereafter, the number of times that each input variable ranked in the top 5 spots across participants’ rankings was retrieved to create Figure 3. Decision tree models appear to have most frequently and highly ranked exercise-related features, followed by environmental variables.

**Figure 4:**
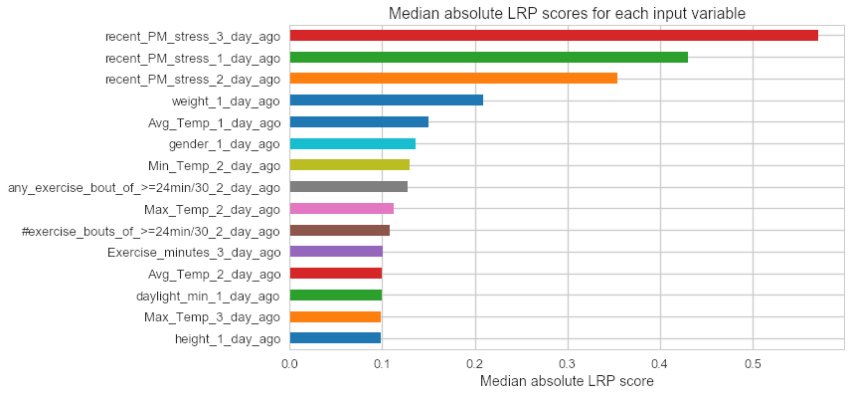
A horizontal bar chart of the 15 predictor variables with the highest median absolute layer-wise relevance propagation scores. LRP: layer-wise relevance propagation.

Figure 4 depicts the relevance scores for the recurrent neural network model, which were derived using LRP. Like the decision tree models, the scores ranged widely, even among participants. To retrieve a metric more comparable with the strictly positive Gini importance values derived from the decision trees, the absolute value of the LRP scores was taken for each participant, followed by the median value for each input variable. The neural network model often ranked the highest, preceding stress self-reports, but there was also significant variety across features such as weather (eg, average or minimum temperature on the day of the stress rating), exercise (eg, number of minutes of exercise or number of exercise bouts the previous day), age, height, and weight.

**Figure 5:**
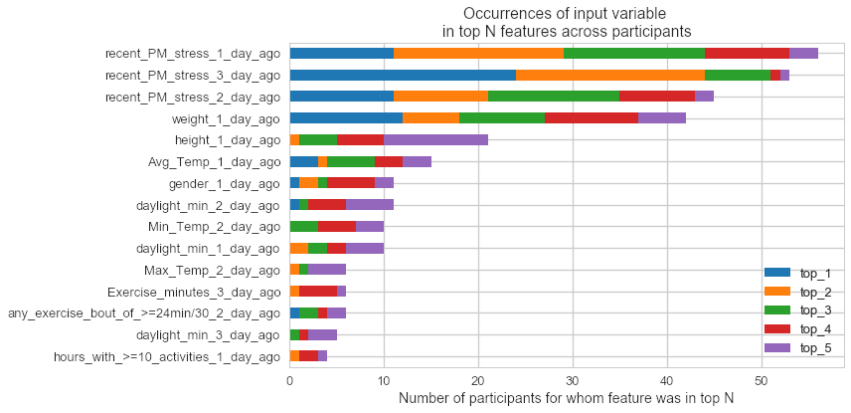
A horizontally stacked bar chart of occurrences of the most frequently appearing predictor variables, and how often they ranked in the top 5 spots across participants’ predictor variable importance rankings from the nomothetic recurrent neural network model.

In Figure 5, as with the decision tree models, the relevance of each input variable was ranked and then counted across participants.

**Figure 6:**
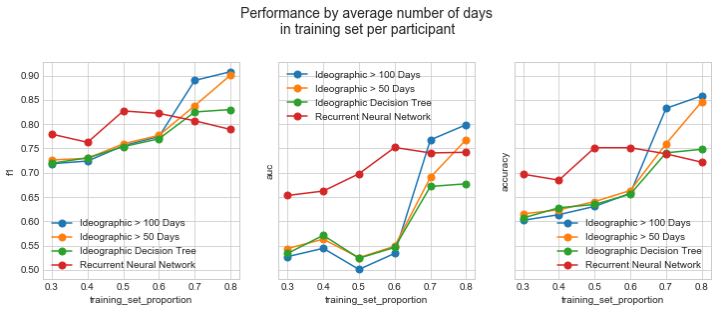
A plot comparing the F1 score, area under the curve, and accuracy across the neural network model and the ideographic models. Training set size varies on the x-axis, and performance of participants with more than 50 valid days and more than 100 valid days is shown separately for the ideographic models. The y-axis is the same in all 3 figures.

Figure 6 depicts the F_1_ score, AUC, and accuracy for the nomothetic recurrent neural network model and the ideographic decision trees across multiple training set sizes. For individuals with fewer than 50 valid days, the nomothetic models generally outperformed or performed comparably as the ideographic ones. Nomothetic model performance and ideographic model performance converged as training set size per participant increased. However, for the 16 individuals with more than 50 valid days and the 9 individuals with over 100 valid days, ideographic performance overtook nomothetic performance. Each dot corresponds to a training set size proportion, ranging from 30% to 80% in increments of 10%. The number of days included in the training set for each proportion across all participants, on an average, was as follows: 17 days for 30%, 22 days for 40%, 27 days for 50%, 33 days for 60%, 38 days for 70%, and 43 days for 80%.

**Figure 7:**
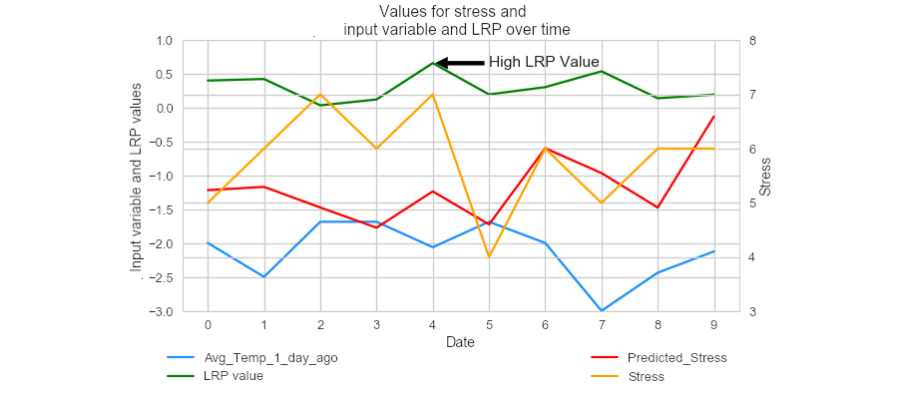
A plot of the actual stress rating, predicted stress rating, layer-wise relevance propagation value, and average temperature for an individual participant, with average temperature standardized to fit on the same graph. LRP: layer-wise relevance propagation.

Using LRP for the nomothetic model, a per-sample feature importance method, allows us to gain insight on what influenced a participant’s predicted stress score for a given day. In Figure 7, a particular participant’s actual stress self-reports, predicted stress self-reports, outside temperature, and the neural network model’s LRP values are depicted across several consecutive days. On day 5, the neural network model assigned a high importance to the average temperature. As shown in the figure, as the average temperature increased (temperature here is standardized to fit on the same scale as the other values), predicted stress and the true stress value increased. Note that in the following graphs, the left-hand y-axis contains the scale for the input variable and the LRP value, and the right-hand y-axis contains the scale for both predicted and true stress rating. The x-axis simply denotes consecutive dates, marked as integers, rather than true dates.

**Figure 8:**
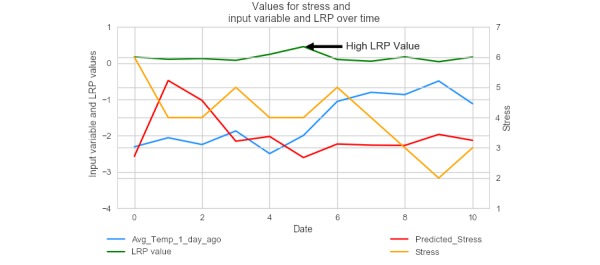
A plot of the actual stress rating, predicted stress rating, layer-wise relevance propagation value, and average temperature for an individual participant, with the average temperature standardized to fit on the same graph. LRP: layer-wise relevance propagation.

For another participant represented in Figure 8, an increase in the maximum temperature on a particular day was associated with a decrease in predicted stress, demonstrating individual-specific response patterns to environmental variables.

**Figure 9:**
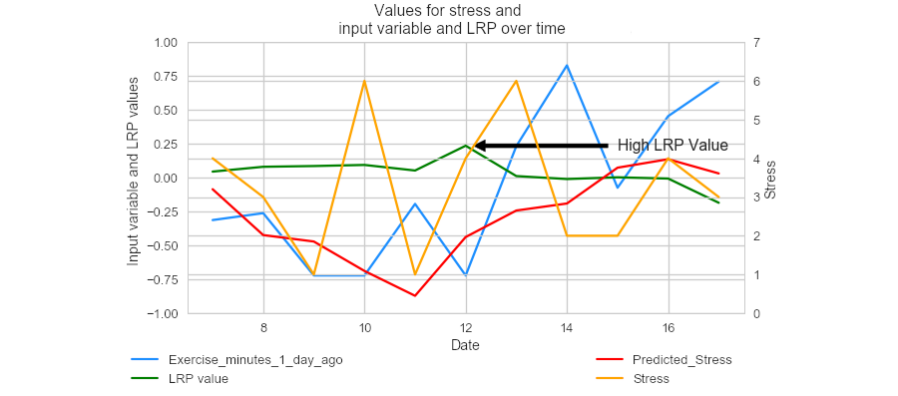
A plot of the actual stress rating, predicted stress rating, layer-wise relevance propagation value, and exercise minutes for a participant, with exercise minutes standardized to fit on the same graph. A lower value for exercise minutes on the previous day was associated with a high layer-wise relevance propagation value and a higher predicted stress rating. LRP: layer-wise relevance propagation.

Exercise, as measured through total minutes or number of separate bouts, was often inversely related with stress rating, where less exercise increased the predicted stress rating (Figure 9).

**Figure 10:**
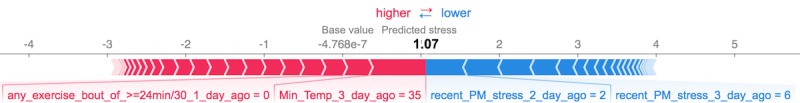
A force plot visualizing the contributions of different input variables to a predicted stress rating for a single participant.

However, there is a risk when interpreting feature importance presented as a time series. Although a variable may carry a high positive influence for a given day, it may be outweighed by a high cumulative negative impact of other variables, causing an improper inference. As such, it is useful to visualize a prediction for a given day not as a time series, but as a force plot of contributions from different variables, allowing us to more easily disentangle their influences. Treating LRP values as approximations for Shapley values, we can retrieve the following force plot depicted in Figure 10 of feature importance for a specific prediction.

Here, the predicted stress self-report value is 1.07. The stress rating 2 days ago of 2 and the stress rating 3 days ago of 6 push the prediction downward. The minimum temperature of 35°F 3 days ago and the absence of any exercise bouts of greater than 24 minutes of moderate-to-vigorous physical activity out of a 30-minute period push the prediction upward.

**Figure 11:**
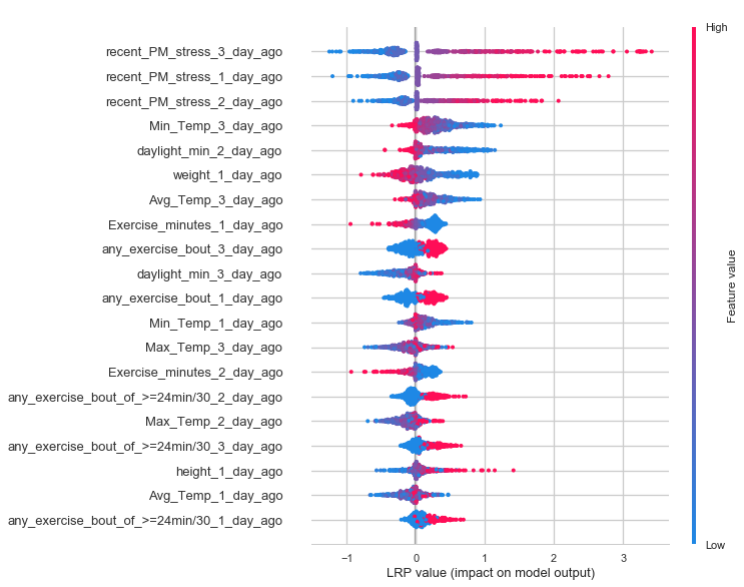
Layer-wise relevance propagation values for each input variable across the entire testing set. Each horizontal line corresponds to a single input variable. LRP: layer-wise relevance propagation.

We can also aggregate these force plots across a set of predictions to understand how our nomothetic neural network model behaves more generally. In Figure 11, each horizontal line displays the LRP values for an input variable, across the entire testing set. The variables are sorted by the sum of the magnitudes of their values, in descending order. The figure shows that higher stress ratings preceding a predicted stress rating typically increase predicted stress, whereas lower minimum temperatures typically increase predicted stress.

## Discussion

### Overview

Many studies have examined relationships among exercise, weather, and stress using both linear and nonlinear approaches, and a mix of self-report questionnaire responses and automatically gathered sensing data. Some have taken an exclusively nomothetic approach; Wang et al [1] used both EMA data and automatically sensed activity and sociability data to explore correlates of stress, depression, and loneliness, but did not use a personalized machine learning approach or investigate individual predictors. Using a large set of meteorological data paired with responses to a self-report questionnaire and a mixed modeling approach, Beecher et al [2] found that increased sun exposure decreased reported distress.

In contrast, other studies have taken an ideographic approach. Tuarob et al [4] used a variety of machine learning techniques and questionnaire data to train ideographic models predicting participant mental states but relied on self-reported input data and did not investigate predictors for each individual. Sharmin et al [14] used sensor data and self-reported stress reports to create personalized visualizations that were then manually examined for temporal trends in stress. Plarre et al [5] trained ideographic decision trees using electrocardiographic and respiration-related data to predict self-reported stress after performing activities in the laboratory setting, such as public speaking or mental arithmetic. Burg et al [6] analyzed the same EMA and exercise data as those used here and estimated ideographic random coefficient mixed models; they found that the influence of exercise on self-reported stress was heterogeneous, as was the effect of self-reported stress on exercise.

Finally, taking an integrative approach and using the same dataset as that used by Burg et al [6] and us, Cheung et al [15] compared the performance of ideographic and nomothetic methods to predict whether an individual will exercise on a given day, again showing that for some, but not all participants, self-reported stress was a predictor of exercise.

In this paper, heterogeneity in the effects of predictors on stress was confirmed, highlighting the value of using an ideographic modeling approach. Further, it was demonstrated that the nomothetic model performs better (ie, is more accurate) than the ideographic model initially, but as data collected accumulates, the performance of the ideographic model equals and then surpasses that of the nomothetic model, providing a motivating example for a “warm start” strategy to leverage the advantages of each modeling approach. Put into practice, one might continuously monitor the performance of a nomothetic model and an ideographic model on a particular individual and adjust the weight of each model’s contribution to the predicted output accordingly.

Retrieving individual predictors yields hypotheses that we can test for a particular participant and, perhaps, act on. For example, if a clinician repeatedly sees that low average temperatures are driving higher predicted stress values, they may be able to recommend a particular intervention for the patient. Of course, not all situations are so easily interpretable, and the relationship of a predictor to the outcome may not be linear. This is both an advantage and a disadvantage, allowing a model to potentially be flexible to multiple climates but limiting the ease of generalizability to other participants, and requires either manual inspection as mentioned above or a more rigorous and automated method of consistent linear predictor detection.

Here, the LRP values from our nomothetic neural network model, and Gini importance values from our ideographic decision trees, suggested different predictors for each individual. The nomothetic neural network model gave preceding stress self-reports higher weight on an average (Figure 10). This may be a result of training the neural network using dropout, in which increasing the weight given to preceding stress reports was an optimal way to manage the heterogeneity of participant response patterns. Notably, Sarker et al [16] reported similar results that stress episodes increase the likelihood of subsequent episodes, although these were within-day data.

Although Figure 4 presents a small sample of individuals and a relatively small dataset overall, it depicts the LRP values for the nomothetic neural network model and motivates discussion of real-world applications of such a model for just-in-time predictions. From this, it seems likely that it is effective to start with a nomothetic model to maximize predictive performance and gain initial insights into the possible correlates of stress before switching to (or increasing the weight of, with an ensemble-type implementation) an ideographic model once enough responses have been collected. This assumes that immediate interventions are necessary, valuable, and worth the cost of potentially introducing bias in the dataset for that individual. Alternatively, predictions could be withheld until sufficient data are collected for ideographic models to be used, but this might come at the expense of disengaging participants during the process of data collection. More generally, adopting a framework, as proposed by Nahum-Shani et al [17], to specify specific proximal outcomes while managing participant engagement is prudent.

### Future Directions and Limitations

There is no current industry or academic standard for retrieving feature importance values from neural networks. Layer-wise relevance propagation, the method used here, has drawbacks of not meeting certain axiomatic properties of ideal feature importance methods [18]. In this case, however, LRP was used because it does not require a baseline, whereas other methods require some reference input for comparison. This instance is not one in which it is immediately clear what that input would be. Other model-agnostic, sensitivity-based approaches exist, such as Locally Interpretable Model Explanations [19]. Regardless of the approach for retrieving feature importance values, we retrieved per-participant predictors from the nomothetic neural network model by taking the median of the absolute value of LRP scores across a subset of the highest-stress events in a participant’s test set. Other strategies may yield features that are more representative of an individual’s stress. Further research in the field may be required to elaborate on strengths and weaknesses of different approaches in terms of interpretability, generalizability, and suitability for inclusion in a clinical decision process.

Next, in this setting, individuals who deviate significantly from the rest of the population may create large gradient updates to a neural network, potentially reducing performance for other individuals. Although this can be mitigated through techniques such as batch normalization and dropout, these strategies, in turn, reduce the degree to which the model can closely fit the data and obscure important differences among individuals. Similarly, individuals contributing a larger number of samples to the training set can bias the model. If an exhaustive hyperparameter search is performed without regularization strategies like L1/L2 penalties, max-norm constraints, dropout, or early stopping, the model may be further overfit to these individuals. As a result, some settings might still be best served by an ideographic approach for safety, but even N-of-1 decision trees may need to be constrained in their complexity to prevent overfit, especially while still acquiring data.

With self-report values, similar to item ratings, variable distributions may be skewed for particular individuals based on their perception of the scale. For example, in this dataset, some individuals never reported their stress to be above a value of 7, despite the scale going up to 10. Examples of these differing response patterns can be found in Figure 1. It is also highly unlikely that responses are missing at random. In fact, these days may be more stressful than the ones reported. The same issue may exist with Fitbit nonwear data. Time series forecasting methods often rely on either excluding or interpolating time windows that have missing data. Here, we do not interpolate missing data and instead, choose to exclude them. As a result, in situations in which self-report data are used, individual response patterns should be taken into account, whether through techniques such as feature engineering or increased data collection.

In our analyses, samples with missing stress ratings prior to the predicted stress rating were excluded. Based on the observed difference in distributions between stress ratings preceded by missing stress ratings, and ones that were not preceded by such ratings, it is possible that the ratings may not be missing at random. Instead, unobserved stress ratings may be indicative of higher stress in some patients. As a result, we primarily explored imputation using a dummy value, but models trained using these values severely underperformed, likely due to the relatively small size of the dataset; therefore, those results were not reported here. Alternatively, missing stress ratings could be imputed using a rule-based approach such as incrementing the most recently observed stress rating. However, for a practical implementation, we believe that with sufficient dataset size, missing stress ratings could be replaced by dummy values to avoid manually biasing predictions and improve generalization. Further, current Fitbit devices and other accelerometers provide continuous heart rate monitoring data, which may provide additional useful predictors and mitigate the effect of missing stress ratings.

Finally, as confidence intervals were not retrieved, we lacked a measure of certainty per prediction. This could be alleviated by using Bayesian neural network or dropout-based methods.

### Conclusions

Through the combination of a nomothetic neural network model, recent advances in retrieving per-sample feature importance, and ideographic decision trees, we show that high predictive performance can be achieved while recognizing individual differences and surfacing personalized predictors of stress. Key predictors in the nomothetic models were typically related to recent stress experience and weather activity. In addition, key predictors in the ideographic models displayed significant heterogeneity but were often weather or exercise related for individuals from whom more data were collected. Environmental variables were also shown to affect stress differently in different participants; for example, high temperatures predicted high stress in one individual but low stress in another. These predictors can be used to provide individuals with insights into what may contribute to their stress, as indicated by Yoon et al [20]. These models can also be operationalized to generate interventions or encouragements just before instances of high stress when the model predicts, with a sufficient degree of confidence, impending stress based on what is expected to be effective for that particular individual. Finally, ideographic models surpassed a nomothetic one after sufficient data collection, supporting the use of a “warm start” model-switching approach. Further work is needed to explore interpretable and repeatable ways to assess personalized predictors in nonlinear settings, as applied to disentangling correlates of stress.

## Appendix

Multimedia Appendix 1 Performance metrics for each model.

## Abbreviations

AUC: area under the curve
EMA: ecological momentary assessment
LRP: layer-wise relevance propagation

## References

[1] Wang R, Chen F, Chen Z, Li T, Harari G, Tignor S (2014). StudentLife: assessing mental health, academic performance and behavioral trends of college students using smartphones.

[2] Beecher ME, Eggett D, Erekson D, Rees LB, Bingham J, Klundt J, Bailey RJ, Ripplinger C, Kirchhoefer J, Gibson R, Griner D, Cox JC, Boardman RD (2016). Sunshine on my shoulders: Weather, pollution, and emotional distress. J Affect Disord.

[3] Steyerberg E (2009). Clinical Prediction Models: A Practical Approach to Development, Validation, and Updating.

[4] Tuarob S, Tucker CS, Kumara S, Giles CL, Pincus AL, Conroy DE, Ram N (2017). How are you feeling?: A personalized methodology for predicting mental states from temporally observable physical and behavioral information. J Biomed Inform.

[5] Plarre K, Raij A, Hossain S, Ali A, Nakajima M, Al-absi M, Ertin E, Kamarck T, Kumar S, Scott M, Siewiorek D, Smailagic A, Wittmers L (2011). Continuous inference of psychological stress from sensory measurements collected in the natural environment.

[6] Burg MM, Schwartz JE, Kronish IM, Diaz KM, Alcantara C, Duer-Hefele J, Davidson KW (2017). Does stress result in you exercising less? Or does exercising result in you being less stressed? Or is it both? Testing the bi-directional stress-exercise association at the group and person (N of 1) level. Ann Behav Med.

[7] Ward DS, Evenson KR, Vaughn A, Rodgers AB, Troiano RP (2005). Accelerometer use in physical activity: best practices and research recommendations. Med Sci Sports Exerc.

[8] (2018). GitHub.

[9] Pedregosa F, Varoquaux G, Gramfort A, Michel V, Thirion B, Grisel O (2011). Scikit-learn: Machine learning in Python. JMLR.

[10] Fawcett T (2006). An introduction to ROC analysis. Pattern Recognition Letters.

[11] Bach S, Binder A, Montavon G, Klauschen F, Müller K, Samek W (2015). On pixel-wise explanations for non-linear classifier decisions by layer-wise relevance propagation. PLoS One.

[12] Shrikumar A, Greenside P, Kundaje A (2017). Learning Important Features Through Propagating Activation Differences.

[13] Lundberg S, Lee S (2017). A unified approach to interpreting model predictions.

[14] Sharmin M, Raij A, Epstien D, Nahum-Shani I, Beck J, Vhaduri S (2015). Visualization of time-series sensor data to inform the design of just-in-time adaptive stress interventions.

[15] Cheung Y, Hsueh P, Qian M, Yoon Sunmoo, Meli Laura, Diaz Keith M, Schwartz Joseph E, Kronish Ian M, Davidson Karina W (2017). Are Nomothetic or Ideographic Approaches Superior in Predicting Daily Exercise Behaviors?. Methods Inf Med.

[16] Sarker H, Hovsepian K, Chatterjee S (2017). From markers to interventions: the case of just-in-time stress intervention. Mobile Health: Sensors, Analytic Methods, and Applications.

[17] Nahum-Shani S, Smith S, Tewari A, Witkiewitz K, Collins L, Spring B (2014). Just-in-Time Adaptive Interventions (JITAIs): An Organizing Framework for Ongoing Health Behavior Support (Technical Report No. 14-126).

[18] Sundararajan M, Taly A, Yan Q (2017). Axiomatic Attribution for Deep Networks. ICML.

[19] Ribeiro M, Singh S, Guestrin C (2016). "Why Should I Trust You?": Explaining the predictions of any classifier.

[20] Yoon S, Schwartz J, Burg M, Kronish Ian M, Alcantara Carmela, Julian Jacob, Parsons Faith, Davidson Karina W, Diaz Keith M (2018). Using Behavioral Analytics to Increase Exercise: A Randomized N-of-1 Study. Am J Prev Med.

