# Supplementary material for: Using Machine Learning to Derive Just-In-Time and Personalized Predictors of Stress: Observational Study Bridging the Gap Between Nomothetic and Ideographic Approaches
Source: J Med Internet Res. 2019 Apr 26;21(4):e12910. doi: 10.2196/12910 (PMC6658264; doi:10.2196/12910)
Supplement: Multimedia Appendix 1 [file jmir_v21i4e12910_app1.pdf]

Table 4 lists performance metrics for each model, grouped by training set proportion and model used. Precision, recall, F_1 score, AUC, and accuracy are performance metrics. Training set size ranges from 30% to 80%. “Ideographic decision tree over 100 days” represents the performance metrics, only for participants who had at least 100 valid days. It is the same for the 50-day model.

Precision	Recall	F1-Score	AUC	Accuracy	Training Set Percentage	Model	Average Number of Days for Training Set Size	Median Number of Days for Training Set Size
74.22%	69.81%	71.95%	53.39%	60.69%	30.00%	Ideographic Decision Tree	16.7215	12
75.76%	69.76%	72.63%	54.40%	61.53%	30.00%	Ideographic Decision Tree Over 50 Days	16.7215	12
76.61%	67.66%	71.86%	52.73%	60.21%	30.00%	Ideographic Decision Tree Over 100 Days	16.7215	12
77.80%	79.75%	78.77%	62.88%	69.75%	30.00%	Random Forest	16.7215	12
78.83%	78.60%	78.72%	64.25%	70.10%	30.00%	Gradient Boosted Decision Tree	16.7215	12
79.90%	76.06%	77.93%	65.32%	69.69%	30.00%	Recurrent Neural Network	16.7215	12
81.09%	65.87%	72.69%	64.71%	65.18%	30.00%	Baseline	16.7215	12
81.20%	80.86%	81.03%	68.21%	73.36%	30.00%	Elastic Net	16.7215	12
82.41%	72.94%	77.39%	68.00%	70.01%	30.00%	Neural Network	16.7215	12
75.35%	69.68%	72.40%	54.44%	61.37%	40.00%	Ideographic Decision Tree Over 100 Days	21.7595	15
76.48%	69.94%	73.06%	57.12%	62.79%	40.00%	Ideographic Decision Tree	21.7595	15
76.96%	69.35%	72.96%	56.32%	62.37%	40.00%	Ideographic Decision Tree Over 50 Days	21.7595	15
77.87%	76.32%	77.09%	62.08%	67.96%	40.00%	Gradient Boosted Decision Tree	21.7595	15
78.15%	80.35%	79.23%	63.16%	70.25%	40.00%	Random Forest	21.7595	15
80.93%	63.42%	71.11%	63.74%	63.61%	40.00%	Baseline	21.7595	15
81.47%	71.66%	76.25%	66.23%	68.47%	40.00%	Recurrent Neural Network	21.7595	15
81.70%	78.65%	80.15%	68.15%	72.48%	40.00%	Elastic Net	21.7595	15
82.33%	76.66%	79.40%	68.55%	71.90%	40.00%	Neural Network	21.7595	15
75.76%	74.96%	75.36%	52.41%	63.47%	50.00%	Ideographic Decision Tree	27.3291	19
77.09%	74.84%	75.95%	52.52%	64.06%	50.00%	Ideographic Decision Tree Over 50 Days	27.3291	19
77.27%	73.91%	75.56%	50.10%	63.07%	50.00%	Ideographic Decision Tree Over 100 Days	27.3291	19
82.41%	82.89%	82.65%	67.54%	74.60%	50.00%	Random Forest	27.3291	19
82.45%	81.45%	81.94%	67.29%	73.80%	50.00%	Gradient Boosted Decision Tree	27.3291	19
84.02%	81.45%	82.71%	69.79%	75.15%	50.00%	Recurrent Neural Network	27.3291	19
84.81%	69.36%	76.31%	67.89%	68.57%	50.00%	Baseline	27.3291	19
85.84%	82.02%	83.89%	72.73%	77.00%	50.00%	Elastic Net	27.3291	19
88.76%	78.09%	83.09%	75.69%	76.79%	50.00%	Neural Network	27.3291	19
78.54%	75.51%	76.99%	54.63%	65.59%	60.00%	Ideographic Decision Tree	32.7215	21

78.85%	76.10%	77.45%	53.40%	65.77%	60.00%	Ideographic Decision Tree Over 100 Days	32.7215	21
79.41%	76.05%	77.69%	54.87%	66.34%	60.00%	Ideographic Decision Tree Over 50 Days	32.7215	21
85.90%	81.67%	83.73%	69.13%	75.75%	60.00%	Random Forest	32.7215	21
86.53%	67.83%	76.05%	66.83%	67.36%	60.00%	Baseline	32.7215	21
88.76%	78.82%	83.49%	73.24%	76.19%	60.00%	Gradient Boosted Decision Tree	32.7215	21
89.80%	81.03%	85.19%	75.62%	78.47%	60.00%	Elastic Net	32.7215	21
90.78%	75.11%	82.20%	75.20%	75.15%	60.00%	Recurrent Neural Network	32.7215	21
91.35%	72.33%	80.73%	75.08%	73.62%	60.00%	Neural Network	32.7215	21
80.69%	81.12%	80.90%	62.87%	71.65%	70.00%	Baseline	38.1646	23
84.52%	80.57%	82.50%	67.17%	74.09%	70.00%	Ideographic Decision Tree	38.1646	23
84.64%	82.94%	83.78%	69.06%	75.96%	70.00%	Ideographic Decision Tree Over 50 Days	38.1646	23
87.35%	84.14%	85.71%	74.69%	79.23%	70.00%	Random Forest	38.1646	23
87.83%	80.15%	83.81%	74.23%	77.08%	70.00%	Gradient Boosted Decision Tree	38.1646	23
88.57%	80.26%	84.21%	75.36%	77.72%	70.00%	Elastic Net	38.1646	23
88.60%	89.38%	88.99%	76.76%	83.25%	70.00%	Ideographic Decision Tree Over 100 Days	38.1646	23
89.16%	73.68%	80.69%	74.07%	73.88%	70.00%	Recurrent Neural Network	38.1646	23
89.57%	76.91%	82.76%	75.69%	76.28%	70.00%	Neural Network	38.1646	23
81.44%	82.15%	81.79%	63.94%	72.81%	80.00%	Baseline	43.2025	28
84.10%	81.92%	83.00%	67.72%	74.82%	80.00%	Ideographic Decision Tree	43.2025	28
88.31%	91.89%	90.07%	76.80%	84.62%	80.00%	Ideographic Decision Tree Over 50 Days	43.2025	28
89.00%	79.90%	84.20%	75.63%	77.71%	80.00%	Elastic Net	43.2025	28
89.00%	78.51%	83.43%	75.18%	76.80%	80.00%	Gradient Boosted Decision Tree	43.2025	28
89.25%	80.59%	84.70%	76.22%	78.35%	80.00%	Random Forest	43.2025	28
90.38%	70.02%	78.91%	74.20%	72.16%	80.00%	Recurrent Neural Network	43.2025	28
90.57%	91.00%	90.78%	79.87%	85.82%	80.00%	Ideographic Decision Tree Over 100 Days	43.2025	28
90.89%	62.22%	73.87%	72.06%	67.27%	80.00%	Neural Network	43.2025	28

Table 4

Performance metrics across all models, grouped by training set size.
